# Acknowledgement to Reviewers of *Non-Coding RNA* in 2018

**DOI:** 10.3390/ncrna5010005

**Published:** 2019-01-10

**Authors:** Non-Coding RNA Editorial Office

**Affiliations:** MDPI, St. Alban-Anlage 66, 4052 Basel, Switzerland

Rigorous peer-review is the corner-stone of high-quality academic publishing. The editorial team greatly appreciates the reviewers who contributed their knowledge and expertise to the journal’s editorial process over the past 12 months. In 2018, a total of 43 papers were published in the journal, with a median time to first decision of 17 days and a median time to publication of 36 days. The editors would like to express their sincere gratitude to the following reviewers for their cooperation and dedication in 2018:
Amodio, NicolaNapierala, MarekAriel, FedericoNatarajan, RamaAtianand, ManinjayNawaz, MuhammadBach, Duc-HiepNazarenko, IrinaBacolla, AlbinoNewbury, Sarah FaithBarteková, MonikaOttaviani, SilviaBeemon, KarenPalancade, BenoîtBerindan-Neagoe, IoanaPalmieri, FerdinandoBidarimath, MallikarjunPanda, AmareshBloom, DavidPapageorgis, PanagiotisBoroviak, KatharinaParashar, DeepakBortoluzzi, StefaniaPfeffer, SébastienBramham, Clive R.Picardi, ErnestoCalin, George A.Pichler, MartinChakrabarti, KausikPillai, RameshChim, JamesPulvirenti, AlfredoCochrane, Dawn R.Puthanveettil, SathyaCosta, ValerioRaimondi, LaviniaCrispim, DaisyReidys, Christian M.Cullen, Bryan R.Renne, RolfCusanelli, EmilioReyes, José C.Czaplinski, KevinRigoutsos, IsidoreDa Costa Martins, Paula A.Riley, Kasandra J.Dakhlallah, DuaaRossetto, Cyprian C.De Bortoli, MicheleRougeulle, ClaireDe Gonzalo-Calvo, DavidRupaimoole, RajeshaDelalle, IvanaSalta, EvgeniaDelihas, NicholasSankaranarayanan, Nehru VijiDhamija, SonamSantulli, GaetanoDiermeier, SarahSchmittgen, ThomasDivoux, AdelineSempere, Lorenzo F.Dziembowski, AndrzejSgrignani, JacopoEarls, LaurieShkumatava, AlenaEl-Hage, NaziraSinclair, JohnEl-Osta, AssamSinnett, DanielEnquobahrie, Daniel A.Slaby, OndrejEsguerra, JonathanSlotkin, KeithFabris, LindaSmalheiser, Neil R.Farnebo, MarianneSmith, Craig A.Ferracin, ManuelaSomarowthu, SrinivasFiszer, AgnieszkaSood, AnilFuentes-Mattei, EnriqueSrikumar, ShabarinathFujita, YuStadler, H. ScottGaetano, CarloStirling, PeterGan, BoyiSzymański, Maciej IejGiroud, MaudeTattikota, Sudhir GopalGoodall, Gregory J.Timmons, LisaGorodkin, JanTollervey, DavidGregory, BrianTonus Marinho, Marco AntonioGromak, NataliaTripp, RalphGutschner, TonyTye, CoraleeGyöngyösi, MariannUchida, ShizukaHan, KookUlitsky, IgorHancock, MeaghanUmansky, SamuilHook, Lauren M.Vandesompele, JoHuang, LeiVentriglia, GiulianaHubé, FlorentVerjovski-Almeida, SergioJackson, MichaelVladimirov, Vladimir I.Jain, VaibhavWahlestedt, ClaesJohnson, RoryWander, Pandora L.Jurak, IgorWang, HsiuyingKapranov, PhilippWatanabe, MariaKhalil, AhmadWeidhaas, JoanneKim, Il-manWeinberg, ZashaKincaid, RodneyWilson, JoyceKöhn, MarcelWilusz, JeffLeclerc, FabriceWittmann, JürgenLi, Pei FengYamamura, SoichiroLu, DongdongYin, CongcongLuna Varo, Rosa MaríaYou, XiaonaMagnus, MarcinYurochko, AndrewMandal, SubhrangsuZhang, ShuxingMedina, PedroZhang, WenbingMeister, GunterZhang, WencaiMiller, W. AllenZhu, Qian-HaoMontgomery, Taiowa A.Zhu, XudongMoran, YehuZiegelbauer, JosephMorris, KevinZielenkiewicz, PiotrMoss, Walter


